# Synthesis of Water-Dispersed Ferrecene/Phenylboronic Acid-Modified Bifunctional Gold Nanoparticles and the Application in Biosensing

**DOI:** 10.3390/ma7085554

**Published:** 2014-07-31

**Authors:** Yun Xing, Lin Liu, Danqing Zhao, Yixin Yang, Xiaoran Chu

**Affiliations:** College of Chemistry and Chemical Engineering, Anyang Normal University, Anyang 455000, Henan, China; E-Mails: yunxinghxx@gmail.com (Y.X.); zdq1201@gmail.com (D.Z.); yxyanghxx@gmail.com (Y.Y.); xrc1202@gmail.com (X.C.)

**Keywords:** gold nanoparticles, ferrecene, boronic acid, biosensors, diols

## Abstract

Phenylboronic acids can form tight covalent bonds with diol-containing biomolecules. In this work, water-dispersed bifunctional gold nanoparticles (AuNPs) modified with ferrecene (Fc)-derivatized peptides and 4-mercaptophenylboronic acids (MBA) (denoted as Fc–MBA–AuNPs) were synthesized and characterized by UV/vis spectrometry and transmission electron microscopy. To demonstrate the application and the analytical merits of the nanoparticles in biosensing, glycoprotein avidin was tested as a model analyte. Specifically, avidin was captured by the biotin-covered gold electrode via the strong biotin-avidin interaction. Then, Fc–MBA–AuNPs were attached by the captured avidin through the formation of tight covalent bonds between the boronic acid moieties of Fc–MBA–AuNPs and the oligosaccharides of avidin. As a result, a detection limit of 0.2 pM was achieved. We believe that the bifunctional nanoparticles would found many applications in amplified detection of diol-containing species by rational design of the surface chemistry of electrode.

## 1. Introduction

Sandwich-type electrochemical absorption biosensors are attractive for a broad range of biomedical, environmental, agricultural, and clinical analyses because they offer multiple advantages, such as high sensitivity, low cost, low power requirement, and high compatibility with advanced micromachining technologies. Sensitivity and noise reduction are crucial for the successful development of such biosensors. Therefore, many efforts have been made to reduce the detection limit by amplifying the signal using various labels, such as functionalized liposomes [1], enzymes [2], carbon-based nanostructures [3,4] and nanoparticles [5,6,7,8]. Among these substances, gold nanoparticles (AuNPs) have been commonly used as labels in diagnostics and detection assays because of their good biological compatibility, high surface-to-volume ratio and excellent conducting capability [5]. Typically, AuNPs are coated with biological elements (e.g., antibody or aptamer) for the recognition of targets and with response molecules (enzymes or redox tags) for the signal output. For example, ferrocene-capped AuNPs have been widely used as the redox markers in electrochemical measurements because of the well-defined and reversible redox reactions of ferrocene [5]. Usually, bifunctional AuNPs modified with ferrocene tags and antibodies or streptavidin can be prepared by the attachment of ferrocenylalkanethiol on the gold surface or by the covalent coupling of ferrocenecarboxylic acid to antibodies or streptavidin [9,10]. These strategies are feasible and convenient, but the loading of a large number of hydrophobic ferrocenylalkanethiol on the AuNPs surface will decrease the dispersion and solubility of the AuNPs suspension. In contrast, modification of antibodies or streptavidin with ferrocenecarboxylic acid will limit the loading amounts of ferrocene tags, resulting in a poor redox peak. Moreover, the utilization of the antibody modified AuNPs might be hindered, especially in resource-poor setting areas, by their high cost and relatively poor stability. Thus, new methods for preparation of ferrecene-capped double-labeled AuNPs are in demand.

Peptides are short chains of amino acid monomers linked by amide bonds. The solubility and chemical properties of peptides are dependent upon their sequence. Moreover, the ferrecene-labeled peptides are inexpensive and readily prepared, and some of them are water-soluble and extremely stable over long storage periods. Because of these properties, ferrecene-labeled peptides site-specifically attached to gold electrodes have been successfully used to detect protease based on the protease-triggered cleavage of peptides [11,12]. Phenylboronic acids are well known to form tight covalent bonds with diol-containing biomolecules [13]. Recent studies have also described the application of boronic acids functionalized nanoparticles in the selective enrichment of glycoproteins, nucleotides and RNA, and demonstrated such nanoparticles as valuable tools for the analysis of glycoproteins due to their unique merits such as ease of production and labeling as well as highly specific binding affinity for diols [14,15,16,17,18,19]. In this work, we reported the synthesis of water-dispersed ferrecene/phenylboronic acid-modified bifunctional AuNPs, and evaluated their analytical merits for the detection of diol-containing species with avidin as a model analyte.

## 2. Experimental Section

### 2.1. Reagents and Materials

4-Mercaptophenylboronic acid (MBA), tris(carboxyethyl)phosphine (TCEP), 6-mercapto-1-hexanol (MCH), bovine serum albumin (BSA), avidin, streptavidin, ferroceneboronic acid (FBA), KH_2_PO_4_ and K_2_HPO_4_ were obtained from Sigma-Aldrich (Changhai, China). The ferrecene (Fc)-derivatized peptide with a sequence of CDK(Fc)DG (denoted as Fc-P) and biotinylated peptide with a sequence of CALNNGK(biotin)G were synthesized and purified by China Peptides Co., Ltd. (Shanghai, China). MBA and Fc-P were dissolved with 0.1 mL methanol, followed by dilution with 0.9 mL 1 mM phosphate-buffered saline solution (PBS, pH 7.2). FBA stock solution was prepared with 200 mM PBS solution (pH 7.2) containing 1% DMSO.

### 2.2. Preparation of Fc-MBA-AuNPs

AuNPs were synthesized using a trisodiumcitrate reduction method as reported previously [20]. Briefly, 5 mL of 38.8 mM trisodium citrate was added into 50 mL of 1 mM HAuCl_4_ boiling solution and the resulting solution was then kept continuously boiling for 30 min until a red solution was obtained. The solution was cooled to room temperature and stored in a refrigerator at 4 °C for use. Modification of AuNPs through the ligand-exchange reaction was performed at room temperature by mixing 1 mL of the as-prepared AuNPs suspension with 1 mL of the PBS solution containing 50 µM TCEP and 5 µM Fc-P. After stirring overnight, 20 µL of MBA at the concentration of 0.5 mM was added into the AuNPs suspension. The unreacted gold surface could be blocked by the small MBA molecules through the formation of an Au-S bond. After reaction for 2 h again, the Fc-P and MBA modified gold nanoparticles (denoted as Fc–MBA–AuNPs) were then thoroughly rinsed with 1 mM PBS to remove the free Fc-P and MBA. The immobilization ability of AuNPs for Fc-P was studied by measuring the free Fc-P in solution with mass spectroscopy. The synthesized Fc–MBA–AuNPs were characterized by Cary 50 UV/vis spectrometry and FEI Tecnai G2 T20 transmission electron microscopy (TEM). The average size of the synthesized AuNPs was also measured with a Zetasizer Nano ZS (Malvern Instruments, Ltd., Worcestershire, UK). The resulting z-average diameter was the average of 3 runs consisting of 11 measurements each.

### 2.3. Procedure for Avidin Detection

The gold disk electrode was polished sequentially with 0.3 μm and 0.05 μm alumina, followed by ultrasonic cleaning in ethanol and water. Then, the electrode was treated electrochemically by cycling the potential in the range of −0.3 and +1.5 V in 0.5 M H_2_SO_4_ solution. The cleaned gold electrode was immersed in a solution of CALNNGK(biotin)G containing 50 μM TCEP and kept overnight at 4 °C. This step was followed by washing the electrode thoroughly with water and soaking it in a 0.1 mM MCH solution for 5 min and a 1% BSA solution for 30 min to block the unreacted gold surface. After the biotin film had been formed, 10 mL of PBS comprising a given concentration of avidin was cast onto the electrode surface for 30 min. Again, the electrode was rinsed with water to remove any non-specifically adsorbed substance. To attach Fc–MBA–AuNPs, the electrode was exposed to 10 μL of the Fc–MBA–AuNPs suspension for 10 min. After the electrode had been rinsed with water, voltammetric determination was performed in a PBS solution containing 50 mM Na_2_SO_4_ on a CHI 660E electrochemical workstation (CH Instruments, Shanghai, China). A platinum wire and an Ag/AgCl electrode were used as the auxiliary electrode and the reference electrode, respectively. Electrochemical impedance spectroscopy (EIS) was collected at the potential of 0.25 V in the frequency range of 0.01 Hz and 500 kHz, and the redox mediator was 1 mM [Fe(CN)_6_]^3−/4−^ (1:1) solution containing 0.1 M KCl. For the investigation of the interaction between avidin and FBA, a glass carbon (GC) electrode with a diameter of 3 mm was used.

## 3. Results and Discussion

### 3.1. Characterization of Fc–MBA–AuNPs

**Figure 1:**
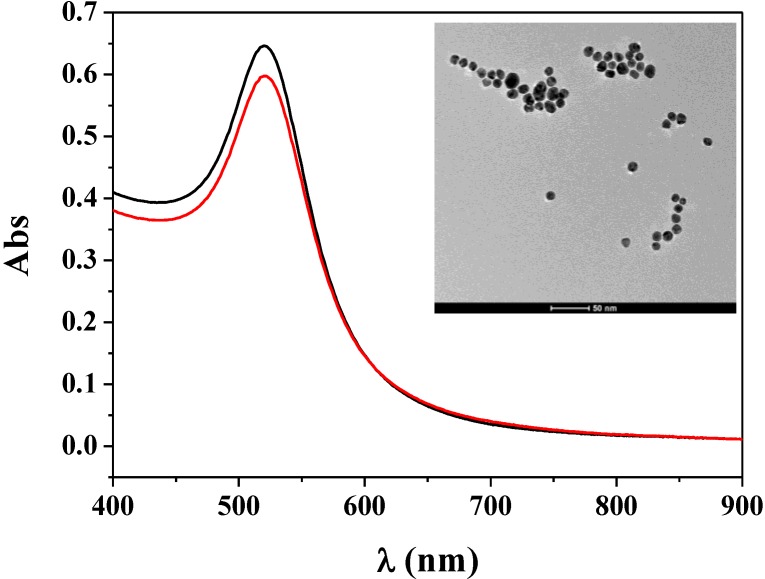
UV/vis absorption spectra of the citrate-stabilized gold nanoparticles (AuNPs) (black curve) and the synthesized ferrecene-derivatized peptides and 4-mercaptophenylboronic acids (Fc–MBA)–AuNPs (red curve). The inset shows the TEM image of the Fc–MBA–AuNPs.

Based on the interaction of boronic acids and cis-diols, boronic acid-modified materials including magnetic particles, polymer nanoparticles and gold nanoparticles have gained increasing popularity for the solid-phase extraction and detection of diol-containing species (e.g., dopamine, glycoproteins and RNA) due to their low bias, convenient enrichment and avoidance of irreversible alterations of analytes [14,17,18,19]. In this work, bifunctional AuNPs labeled with boronic acid moieties and electrochemically active ferrecene tags were designed and prepared through the ligand-exchange reaction. The synthesized Fc–MBA–AuNPs were characterized by UV/Vis spectroscopy. As shown in Figure 1, the Fc–MBA–AuNPs suspension displayed a characteristic UV/Vis absorption spectrum with a plasmon band at 520 nm (red curve). Such absorption was ascribed to the surface plasmon resonance of the AuNPs (black curve). Note that the loading of MBA on AuNPs could cause the aggregation and color change of AuNPs due to the formation of a planar 6-membered boroxine ring with the elimination of three water molecules from three MBA molecules [21]. We found that the Fc–MBA–AuNPs were stable and monodisperse (see the TEM image in the inset), indicating that the modification of AuNPs with Fc-P peptides inhibited the formation of a planar 6-membered boroxine ring between three MBA molecules. We also found that the average loading number of Fc-P per Au nanoparticle was around 780 by measuring the free peptide in solution with mass spectroscopy. Such high loading implied that the analytical method based on the signal amplification of Fc–MBA–AuNPs would be very sensitive. Additionally, no free MBA was found in the solution, demonstrating that all MBA molecules were absorbed onto the unreacted gold surface. Thus, one Fc–MBA–AuNP contained ~1000 MBA molecules, which will facilitate the capture of Fc–MBA–AuNPs by the anchored glycoproteins. Furthermore, we found that the z-average size of the Fc–MBA–AuNPs measured by a zetasizer was 26.7 nm, which is larger than that of bare AuNPs (22.1 nm). Note that the size of the AuNPs measured by the zetasizer was larger than that obtained by TEM (12.8 nm). This can be attributed to the formation of hydration layer around the AuNPs in the former case [22].

### 3.2. Application of Fc–MBA–AuNPs in Biosensing

**Figure 2:**
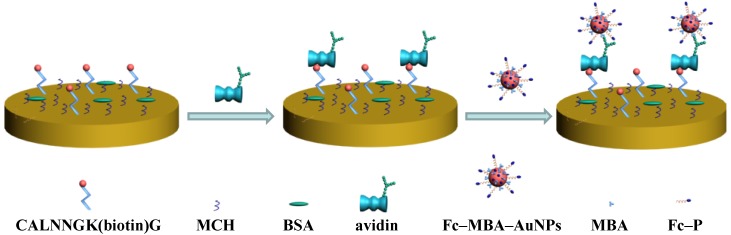
Schematic illustration for avidin detection using bifunctional Fc–MBA–AuNPs.

**Figure 3:**
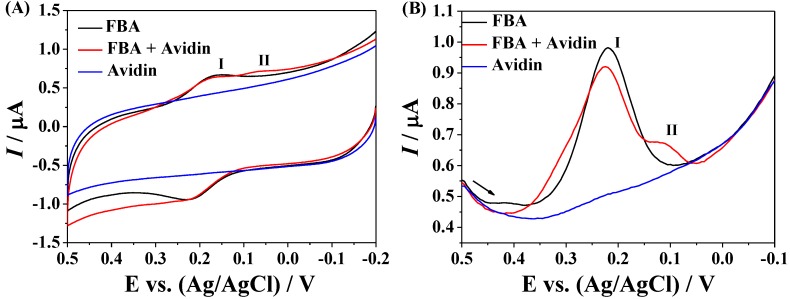
(**A**) Cyclic voltammograms (CVs); and (**B**) differential pulse voltammograms (DPVs) collected at glass carbon (GC) electrode in the solution of ferroceneboronic acid (FBA), avidin and FBA/avidin. The final concentrations of FBA and avidin were 20 and 2 μM, respectively.

Glycoproteins are proteins that contain oligosaccharide chains covalently attached to polypeptide side-chains. They play key roles in a wide variety of biological processes, including cellular adhesion, cell signaling, cell-cell communication and immune response [23]. The changes in the level of glycoproteins have also been demonstrated to be associated with many diseases, such as inflammation and cancers [24,25]. To evaluate the analytical performances of Fc–MBA–AuNPs, we demonstrated their application in detection of less expensive glycoprotein avidin. The sensing principle of the method was shown in Figure 2. The biotinylated peptides were immobilized on gold electrode through the formation of Au-S bond. The surface coverage of the immobilized peptides, estimated electrochemically using Fe(CN)_6_^3−^ [26], was found to be around 13.7 pmol/cm^2^. To avoid the nonspecific adsorption of avidin on electrode, the unreacted gold surface was blocked by MCH and BSA. Then, avidin was captured by the sensing electrode by the strong biotin–avidin interaction [27], which allowed Fc–MBA–AuNPs to be anchored through the interaction between boronic acids on AuNPs and carbohydrate moieties of avidin. Previously, it has been reported that the formation of boronate ester can induce the change in the redox potential of ferroceneboronic acid (FBA) [28]; also, FBA has been used as the redox probe to recognize the glycated hemoglobin immobilized on ZrO_2_-nanoparticles modified electrode [29]. Herein, we first investigated the interaction between boronic acid and avidin with FBA as the redox probe. As shown in Figure 3A, FBA exhibits a couple of redox waves with the oxidation potential at 0.23 V and the reduction potential at around 0.17 V (peak I). After the addition of avidin, a new reduction peak at around 0.08 V (peak II) appeared, which was further confirmed by the differential pulse voltammograms (Figure 3B). These results indicated that it is feasible to detect avidin with boronic acids as the recognition units based on the boronic acid-carbohydrate interaction.

### 3.3. Amplified Voltammetric Determination of Avidin

**Figure 4:**
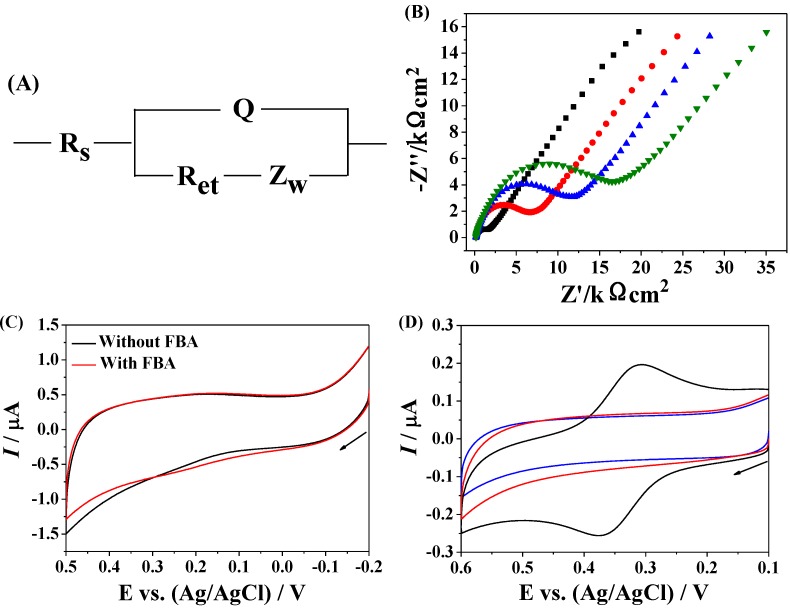
(**A**) The fits with the Randles equivalent circuit; (**B**) electrochemical impedance spectroscopy (EIS) of bare (black curve), biotin-covered (red curve), biotin/MCH/BSA-covered (blue curve), and biotin/MCH/BSA/avidin-covered (olive curve) gold electrode; (**C**) CVs of the biotin/MCH/BSA/avidin-covered electrode before (black curve) and after (red curve) incubation with FBA solution; (**D**) CVs of the biotin/MCH/BSA-covered electrode with (black curve) and without (red curve) the capture of avidin, followed by the attachment of Fc–MBA–AuNPs. The blue curve in panel D corresponds to that with streptavidin in place of avidin. The concentration of avidin was 157 pM, and the arrow indicates the scan direction.

Electrochemical impedance spectroscopy can be used to examine the property of self-assembled monolayers (SAMs) including surface coverage and monolayer composition, and the conduction ability of electrode. To demonstrate the immobilization of biotinylated peptides and the capture of avidin, we determined the change in the impedance of gold electrode. A modified Randles equivalent circuit was used to fit the impedance spectra and to determine electrical parameters for each step. As shown in Figure 4A, the circuit included the electrolyte resistance between working and reference electrodes (Rs), the Warburg impedance (Zw), a constant phase element (Q) representing the double layer capacitance for an unmodified electrode or the capacitance of the SAMs for the modified electrodes and the electron-transfer resistance (Ret). As shown in Figure 4B, the bare gold electrode showed a small electron transfer resistance (black curve), demonstrating a fast electron transfer process. The increase in the Ret implied the immobilization of biotinylated peptides (red curve). The block of unreacted gold surface by the MCH and BSA induced an increase in the Ret (blue curve). The results are understandable since the block of gold surface could retard the interfacial electron transfer kinetics of [Fe(CN)_6_]^3−/4−^ anions. Furthermore, we found that incubation of the sensing electrode with avidin solution led to a small increase in the Ret (olive curve), indicating that avidin was captured by the biotin-covered electrode. We first attempted to detect the captured avidin using FBA probes (Figure 4C). As a result, no obvious change was observed at the avidin modified electrode before and after incubation with FBA. In turn, a couple of well-defined voltammetric wave was observed with Fc–MBA–AuNPs as the redox probes (black curve in Figure 4D), demonstrating that the electrochemical signals were greatly amplified by AuNPs. The control CV (red curve in Figure 4D) was acquired in the absence of avidin. The voltammetric peak currents dropped to a background level, indicating that the attachment of Fc–MBA–AuNPs is dependent on the captured avidin. For comparison, the same procedure was implemented with electrodes covered with streptavidin, a biotin-binding protein lacking any carbohydrate modification. The absence of any discernible peaks in the blue curve indicates that binding of Fc–MBA–AuNPs is strictly dependent on the formation of boronate ester between MBA and carbohydrate moieties of avidin. Thus, the proposed method based on the signal amplification of Fc–MBA–AuNPs was feasible for the recognition and detection of diol-containing species.

### 3.4. Dependence on Avidin Concentration

**Figure 5:**
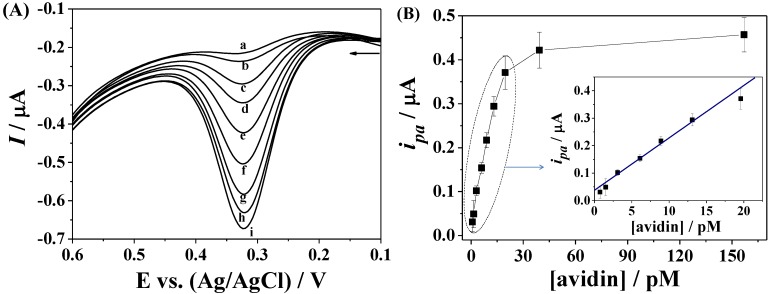
(**A**) DPVs of the sensing electrode at different concentrations of avidin (a–i: 0.75, 1.5, 3.1, 6.2, 8.9, 13.1, 19.6, 39.3 and 157 pM); (**B**) plots of the *i_pa_* against the avidin concentration (0.75–19.6 pM).

Differential pulse voltammetry can decrease the background charging currents and in turn increase the detection sensitivity. Thus, we evaluated the sensitivity of the proposed method for avidin detection using differential pulse voltammetry. Figure 5A shows the typical DPVs of the biotin-covered electrode after incubation with different concentrations of avidin, followed by the attachment of Fc–MBA–AuNPs. The oxidation currents (*i_pa_*) increase linearly with the concentrations of avidin ranging from 0.75 to 19.6 pM, and begin to level off beyond the concentration Figure 5B. The linear regression equation can be expressed as *i_pa_* (µA) = 0.038 + 0.019 [avidin] (pM) (*R*^2^ = 0.99). The detection limit (3 s) of the method was estimated to be 0.2 pM, which is lower than those obtained by the amplification of ferrocene/antibody-labeled AuNPs/chitosan nanocomposites [30] and ferrocene-capped AuNPs/streptavidin conjugates [10]. The lower detection limit is attributed to the high loading of ferrocene tags and the electrocatalytic property of AuNPs.

## 4. Conclusions/Outlook

In summary, bifunctional AuNPs coated with boronic acid moieties for the recognition of diols and with ferrecene redox tags for the signal output were synthesized. To demonstrate the application and the analytical merits of the nanoparticles in biosensing, glycoprotein avidin was tested as a model analyte. The detection limit of 0.2 pM is lower than those achieved by the amplification of ferrocene/antibody-labeled AuNPs/chitosan nanocomposites and ferrocene-capped AuNPs/streptavidin conjugates. Moreover, compared to other sandwich-type electrochemical biosensors, the presented approach obviates the need of expensive antibodies as the recognition units of targets. We believe that the bifunctional nanoparitcles would find many applications in the detection of diol-containing species including glycoproteins and RNA by rationally designing the surface chemistry of electrodes.
